# Molecular mechanism of Danxiong Tongmai Granules in treatment of coronary heart disease

**DOI:** 10.18632/aging.205845

**Published:** 2024-05-21

**Authors:** Jiahao Ye, Ruiping Yang, Lin Li, Senjie Zhong, Ruixue Jiang, Zhixi Hu

**Affiliations:** 1 College of Chinese Medicine, Hunan University of Chinese Medicine, Changsha, Hunan 410208, China; 2 Basic Medical Sciences College, Hubei University of Chinese Medicine, Wuhan, Hubei 430065, China; 3 The First Affiliated Hospital of Guangzhou University of Chinese Medicine, Guangzhou University of Chinese Medicine, Guangzhou, Guangdong 510405, China

**Keywords:** Danxiong Tongmai Granules, coronary heart disease, network pharmacology, molecular docking, molecular dynamics simulation

## Abstract

Background: Danxiong Tongmai Granules (DXTMG) are widely utilized in treating coronary heart disease (CHD) in China. This study aims to explore the molecular mechanisms underlying the therapeutic effects of DXTMG on CHD by employing a network pharmacology approach, complemented with experimental validation.

Methods: Traditional Chinese Medicine (TCM) compounds and targets were identified via searches in the BATMAN-TCM database, and the GeneCards database was used to obtain the main target genes involved in CHD. We combined disease targets with the drug targets to identify common targets. The “TCM-compound-target” network was plotted using Cytoscape 3.7.2 software and a protein-protein interaction (PPI) network was constructed using the STRING database from which core targets were obtained. Gene ontology (GO) function analysis and Kyoto Encyclopedia of Genes and Genomes (KEGG) pathway enrichment analysis were performed for common drug-disease targets using R Version 4.0.4 (64 bit) software. Molecular docking of core protein-small molecule ligand interaction was modeled using AutoDock software. A molecular dynamics simulation was conducted on the optimal protein-small molecule complex identified through molecular docking, using Amber18 software. The rat model for myocardial ischemia was established through pre-gavage administration of DXTMG, followed by dorsal hypodermic injection of isoprenaline. Myocardial tissues from the rats were analyzed using hematoxylin and eosin (HE) staining and Masson’s trichrome staining. Relevant targets were validated by enzyme-linked immunosorbent assay (ELISA) and immunohistochemistry.

Results: 162 potential targets of DXTMG involved in CHD were identified. These included INS, ALB, IL-6 and TNF according to PPI network studies. GO enrichment analysis identified a total of 3365 GO pathways, including 3049 biological process pathways (BP) concerned with the heart and circulatory system; 109 cellular component (CC) pathways, including cation channels and membrane rafts and 207 molecular function (MF) pathways related to receptor ligands and activators. KEGG analysis revealed a total of 137 pathways (*P* < 0.05), including those related to AGE-RAGE signaling associated with diabetic complications, fluid shear stress and atherosclerosis. The results of molecular docking and molecular dynamics simulations demonstrated the robust binding affinity between the compounds and target proteins. Animal experiment findings indicated that, compared with the model group, the DXTMG group effectively ameliorated inflammation and fibrosis in rat myocardial tissues, reduced LDH, cTn-I, and MDA levels (*P* < 0.05, *P* < 0.01), elevated SOD and GSH-PX levels (*P* < 0.05), and reduced the percentage of positive area for IL-6 and TNF-α (*P* < 0.05).

Conclusion: This study preliminarily suggests that DXTMG can modulate oxidative stress, inflammation response, and cardiomyocyte regulation, thereby mitigating the onset and progression of CHD.

## INTRODUCTION

Coronary heart disease (CHD) occurs when atherosclerosis causes luminal stenosis or occlusion, leading to insufficient blood supply to the coronary artery and failure to meet the demands of myocardial metabolism [1]. According to the American Heart Association, 15.5 million adults in the United States suffered from CHD in 2016 [2]. CHD has a serious impact on the patient’s quality of life and is characterized by poor prognosis causing high morbidity and mortality rates. Therapeutic approaches commonly taken by Western medicine have many side effects, especially during long-term administration. For example, a 4-month course of statins may contribute to liver damage with raised ALT and AST levels and the development of hepatocyte necrosis, liver fibrosis and increased inflammatory responses [3]. Moreover, there is an increased risk of new-onset diabetes [4]. By contrast, Traditional Chinese Medicine (TCM) often has multi-target and multi-pathway synergistic effects and low incidence of toxic side effects. Thus, TCM has great potential for the treatment of CHD.

Danxiong Tongmai Granules (DXTMG) have gained increasing popularity for treating CHD. Yao et al. [5] treated 171 patients with angina pectoris due to coronary heart disease using DXTMG. The effective rate for exertional angina treatment was 97.28%, while for spontaneous angina, it was 95.83%. Following DXTMG administration, there was a significant reduction in nitroglycerin dosage, and partial discontinuation of the drug was observed. Furthermore, Tang et al. [6] demonstrated that DXTMG effectively lowers systolic and diastolic blood pressure, reduces heart rate, enhances the aorta’s elasticity index, and contributes to the protection against cardiovascular and cerebrovascular diseases. Additionally, Shi et al. [7] investigated the effects of DXTMG on hypertensive patients. Platelet activation levels were measured using flow cytometry, revealing a significant reduction in PACI^+^% and CD62P^+^% levels post-treatment, approaching normal levels. Moreover, improvements in blood lipids and glucose levels were observed. To date, research on DXTMG has primarily focused on clinical observation, with limited progress in elucidating its molecular mechanisms.

**Figure 1:**
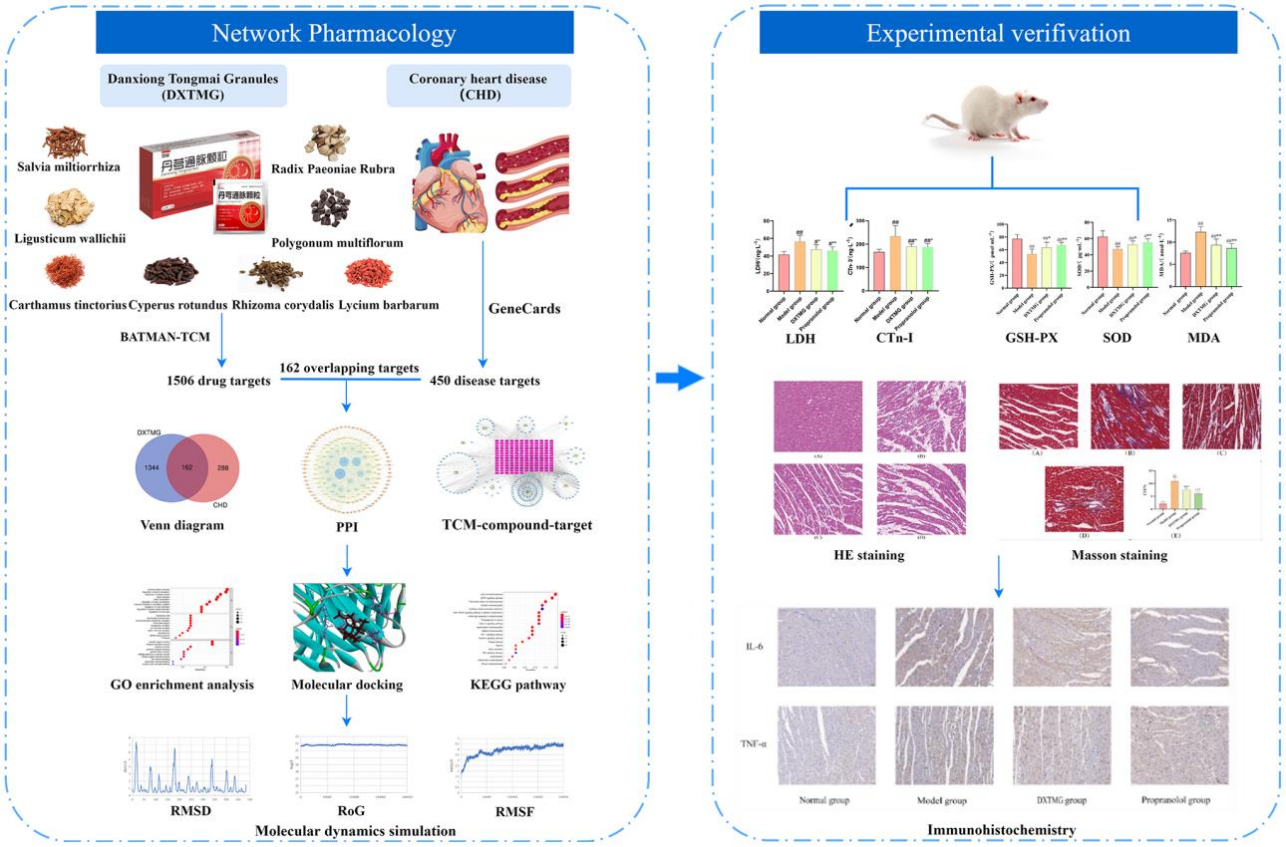
Workflow chart of DXTMG for the potential treatment of coronary heart disease based on network pharmacology.

Network pharmacology is a novel pharmaceutical research method for the systematic analysis of “multi-component-multi-target-multi-pathway” characteristics and has been applied to TCM [8, 9]. Molecular docking is an approach whereby small ligand molecules are modeled within the receptor binding site so that spatial interactions may be explored. Using flexible and semi-flexible docking, receptor-ligand interactive forces may be assessed enabling prediction of binding mode and affinity [10]. Molecular dynamics allows the simulation of various ligand and receptor movements via Newtonian mechanics to assess stability and flexibility. During the present study, core target receptors and ligands were identified through network pharmacology, and interaction sites and binding characteristics were analyzed using molecular docking and molecular dynamics simulations. Finally, the relevant targets were validated through animal experiments to explore the mechanism of DXTMG in treating CHD. The flowchart is shown in Figure 1.

## METHODS

### Chemical compounds and target genes

The components of DXTMG comprise Radix Paeoniae Rubra, Ligusticum wallichii, Salvia miltiorrhiza, Polygonum multiflorum, Rhizoma corydalis, Cyperus rotundus, Carthamus tinctorius, and Lycium barbarum. Parameters set in the BATMAN-TCM database include a Score cutoff of the system-recommended minimum score of 20 and an adjusted *P*-value threshold value of 0.05 [11].

### Targets of CHD

A total of 450 CHD target genes were obtained from the GeneCards database (https://www.genecards.org/) using “coronary heart disease” as the keyword and the criterion of Score >30 [12]. Target genes common to both DXTMG and CHD were identified using the Venny diagram online tool (http://bioinformatics.psb.ugent.be/webtools/Venn/), and a Venny diagram was generated.

### Construction of “TCM-compound-target” network

The effective compounds and drug targets were imported into Cytoscape 3.7.2 software and a “TCM-compound-target” network diagram was plotted [13]. The built-in tool, Network Analyzer, was used to analyze the associations.

### Construction of “protein-protein” interaction (PPI) network

Common target proteins were imported into the STRING database (https://www.string-db.org/) [14] to construct a PPI network. The species was set to “Homo sapiens”, and the confidence level was set to >0.4. Unconnected nodes were hidden, and the PPI network diagram was plotted using Cytoscape 3.7.2 software.

### Gene ontology (GO) and Kyoto Encyclopedia of Genes and Genomes (KEGG) analysis

GO and KEGG analysis were conducted using R package of clusterProfiler [15]. GO enrichment analysis covered biological process (BP), molecular function (MF) and cellular component (CC). The top 10 GO and the top 20 KEGG pathways were identified (*p* < 0.05).

### Molecular docking

The five compounds with the highest scores in the “TCM-Compound-Target” network diagram and the four core proteins with the highest scores in the PPI network underwent molecular docking analysis using AutoDock software [16]. The ligand file was downloaded from the PubChem database (https://pubchem.ncbi.nlm.nih.gov), and energy minimization of the ligand was performed using Chem3D software. Subsequently, the ligand was saved in pdb format, and the charge was calculated using AutoDockTools 1.5.6 software and saved in pdbqt format. The 3D crystal structure of the core target was obtained from the RCSB PDB database (http://www.rcsb.org/) and saved in pdb format. Parameters related to the active pocket were determined, and semi-flexible molecular docking was conducted to calculate the “ligand-receptor” binding energy. Finally, the best docking model was generated using Discovery Studio 4.5 software. Binding energies were categorized as follows: >−5 for initial binding, >−7 for stronger binding, and >−9 for very strong binding [17]. The molecular docking results were calculated three times, and the mean value and standard deviation were computed and expressed as “mean value ± standard deviation”.

### Molecular dynamics simulation

The Amber18 software package was used to perform molecular dynamics simulation on the protein (target) and small molecule ligand (compound) obtained by molecular docking. ff14SB and general Amber force field (GAFF) parameters were used for proteins and small molecule ligands, respectively, and the AM1-BCC atomic charge was calculated using the ANTECHAMBER module. The protein-ligand complex was loaded into the leap module, and hydrogen atoms and antagonist ions were automatically added to neutralize the charge. The TIP3P explicit water model was selected, and periodic boundary conditions were set. The workflow of molecular dynamics simulation included energy minimization, heating, equilibration and production dynamics. Heavy atoms of proteins were constrained, and water molecules were subjected to 10,000 steps of energy minimization (including 5000-step steepest descent method and 5000-step conjugate gradient method). The system was slowly heated to 300 K within 50 ps and equilibrated for 50 ps under the NPT ensemble. Molecular dynamics simulation was conducted on the system under the NPT ensemble for 200 ns (25,000 steps in total) with a time step of 2 fs. The trajectory data were saved every 10 ps, followed by analysis using the CPPTRAJ module. The free energy of binding for ligands and proteins was calculated using the MMPBA.py module.

### Experimental validation

#### Animals

Forty male SD rats of SPF grade, aged 6–8 weeks and weighing 200 ± 20 g, were provided by Hunan Slaughter Kingda Laboratory Animal Co. Ltd, under Laboratory Animal Use License No. SCXK (Hunan) 2019-0004. The rats were bred at the Animal Experiment Center of Hunan University of Traditional Chinese Medicine and fed a standard diet. This experiment was reviewed and approved by the Experimental Animal Ethics Committee of Hunan University of Traditional Chinese Medicine, with Ethics Approval No. LL202310250002. The experimental design and procedures adhered to the Hunan Experimental Animal Regulations and the National Institutes of Health Guidelines for the Ethical Use of Animals.

#### Drugs

Danxiong Tongmai Granules (Sichuan Enwei Pharmaceutical Co., Ltd., 5 g/bag, Lot No. Z20010180); propranolol (Jiangsu Yabang Apsen Pharmaceutical Co., Ltd., Lot No. E230516); isoprenaline (Shanghai Aladdin Biochemical Technology Co., Ltd., Lot No. I129810).

#### Reagents

Rat Superoxide Dismutase (SOD) ELISA Kit, Rat Lactate Dehydrogenase (LDH) ELISA Kit, Rat Malondialdehyde (MDA) ELISA Kit, Rat Cardiac Troponin I (cTn-I) ELISA Kit, Rat Glutathione Peroxidase (GSH-PX) ELISA Kit (Hunan Alfon Bio Co., Ltd., batch no. Ltd, lot number: AF20230731); TNF-α Primary Antibody Kit (Aifang Bio, lot number: AF06294); IL-6 Primary Antibody Kit (Xavier Bio Co., Ltd, lot number: GB11117); HRP-Polymer Anti-Rat Secondary Antibody Kit (Aifang Bio, lot number: AFIHC002); HRP-Polymer Anti-Rabbit Secondary Antibody Kit (Aifang Bio, Lot No.: AFIHC003); anhydrous ethanol, xylene, n-butanol, neutral gum (Sinopharm Chemical Reagent Co., Ltd., Lot Nos. 100092683, 10023418, 100052190, and 10004160, respectively); Masson’s staining solution kit (Servicebio, Lot No.: G1006); Hematoxylin Differentiation Solution, Hematoxylin Blue Return Solution, Citrate Buffer (Hunan Aifang Biotechnology Co., Ltd, lot numbers: AFIHC019, AFIHC020, AFIHC009); BSA (Servicebio, lot number: G5001).

#### Instruments

Dehydrator (Wuhan Junjie Electronics Co., Ltd., model: JJ-12J); embedding machine (Wuhan Junjie Electronics Co., Ltd., model: JB-P5); pathology sectioning machine (Shanghai Leica Instruments Co., Ltd., model: RM2016); orthogonal optical microscope (Nikon, Japan, model: Nikon Eclipse E100); enzyme labeling machine (Labsystems Multiskan MS, Model: Model 352).

#### Grouping, modeling and drug administration

Forty male SD rats aged 6–8 weeks with SPF grade, weighing 200 ± 20 g, were used. After 3 days of acclimatization, the rats were randomly divided into the following groups: normal group, model group, propranolol group (10 mg/kg), and DXTMG group (2.7 g/kg), each consisting of 10 rats. The corresponding dose of the drug (10 mL/kg) was administered by gavage in each treatment group, while the normal and model groups received an equal volume of saline once daily for 7 consecutive days. Two hours after drug administration on the 6th day, isoproterenol (85 mg/kg) was subcutaneously injected into the backs of the rats in the propranolol group, the DXTMG group, and the model group for 2 consecutive days. An equal volume of saline was injected subcutaneously into the normal group [18]. Twenty-four hours after modeling completion, the rats were anesthetized again, blood was collected from the fundus venosus plexus, the rats were euthanized, and their hearts were extracted. The hearts were fixed with 4% paraformaldehyde, excess tissue was excised on ice, leaving only the left ventricle, which was washed with saline and stored in a −80°C freezer for subsequent detection of relevant indexes.

#### Serum LDH and cTnI level detection

The rats’ blood was allowed to stand at room temperature for 2 hours, centrifuged at 4°C and 4000 rpm for 15 minutes, and the supernatant was extracted. Serum LDH and cTnI levels were detected using a microplate assay.

#### HE staining

Myocardial tissue was fixed with 4% paraformaldehyde, embedded in conventional paraffin, and sectioned into 3 μm thick slices. Subsequently, the sections were dewaxed to water and stained with hematoxylin for 3~5 minutes. Differentiation was achieved with hydrochloric acid in an aqueous solution followed by blue return using ammonia. The sections were then dehydrated with a gradient of ethanol and stained with eosin staining solution for 5 minutes. Finally, the sections were dehydrated, sealed, and images were captured using a digital section scanner to observe tissue inflammation, degeneration, and necrosis.

#### Masson staining

Following dewaxing, sections with a thickness of 3 μm were immersed in potassium dichromate at 60°C for 30 minutes. Subsequently, they were stained with ferric hematoxylin for 3 minutes, washed with water, and immersed in Lichun red acidic magenta solution for 5–10 minutes. Afterward, the sections were placed in phosphomolybdic acid aqueous solution for 1–3 minutes and then transferred to aniline blue staining solution for 3–6 minutes. Differentiation was carried out using 1% icosahexaenoic acid, followed by transparent sealing and image acquisition.

#### Detection of SOD, GSH-Px activity and MDA level in myocardial tissue

An appropriate amount of frozen myocardial tissue was taken, weighed, homogenized, and centrifuged at 4°C and 5000 rpm for 10 minutes. The supernatant was extracted, and the SOD, GSH-Px activity, and MDA level in myocardial tissue were detected according to the instructions of the kit.

#### Immunohistochemistry method to detect myocardial tissue inflammatory factors IL-6, TNF-α protein

Tissue Inflammatory Factors IL-6 and TNF-α Protein Myocardial tissue paraffin sections were dewaxed to water, and repair solution was added into the autoclave, boiled for 2 minutes, and naturally cooled at room temperature. Subsequently, the sections were removed, and after natural cooling, they were placed in PBS (pH 7.4) in a decolorizing shaker for three washes, each lasting 5 minutes. The sections were then immersed in 3% hydrogen peroxide solution and incubated at room temperature in the dark for 15 minutes, followed by three washes, each for 5 minutes. In the histochemical circle, 3% BSA was added to evenly cover the tissues and incubated at room temperature for 30 minutes. The primary antibodies (IL-6 primary antibody prepared in PBS at a dilution of 1:100, TNF-α primary antibody prepared in PBS at a dilution of 1:200) were incubated overnight at 4°C. After three washes, each for 5 minutes, the sections were slightly dried, and the secondary antibody was added dropwise into the circle. The sections were then incubated for 50 minutes at room temperature, washed with PBST, and color development was initiated by DAB. The color development was terminated with PBST, and the nuclei were stained with hematoxylin for 2 minutes. After washing, the blue color was restored to the nucleus by reverse deconjugation. The sections were then incubated at room temperature, and the cells were stained with hematoxylin for 2 minutes, washed with water, warmed with warm water, re-blued, reverse dewaxed, hydrated, sealed with clear neutral gum, and photographed. IL-6 and TNF-α positive cells appeared as yellow or brownish-yellow granules in the cytoplasm, while the Normal group did not show such staining. ImageJ software was used for analysis.

### Statistical analysis

Statistical analysis was conducted using SPSS 24.0 software, and the data were expressed as $\bar{x}\pm \text{s}.$ Group comparisons were performed using the *t*-test for two-group comparisons and one-way ANOVA for multiple-group comparisons. *P* < 0.05 indicates that the difference is statistically significant.

### Data availability

All data generated or analyzed during this study are available from public databases, published articles and Supplementary Materials.

## RESULTS

### Screening of active pharmaceutical ingredients and targets

Compounds from DXTMG retrieved from the BATMAN-TCM database included 9 compounds from Radix Paeoniae Rubra, 91 from Ligusticum wallichii, 39 from Salvia miltiorrhiza, 16 from Polygonum multiflorum, 37 from Rhizoma corydalis, 12 from Cyperus rotundus, 22 from Carthamus tinctorius and 1 from Lycium barbarum. Seven replicates were removed leaving 220 compounds in total (Supplementary Table 1). In addition, a total of 11,121 drug targets were retrieved, including 127 targets of Radix Paeoniae Rubra, 2163 of Salvia miltiorrhiza, 4970 of Ligusticum wallichii, 300 of Polygonum multiflorum, 1533 of Rhizoma corydalis, 1074 of Cyperus rotundus, 913 of Carthamus tinctorius, and 41 of Lycium barbarum. Removal of replicates left a total of 1506 targets.

### Screening of CHD target genes and construction of “TCM-Disease” intersect targets

**Figure 2:**
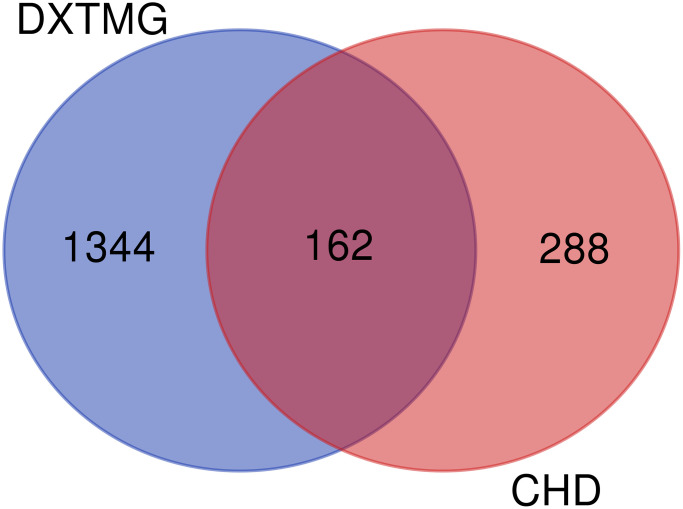
Venny diagram of DXTMG (blue) and CHD targets (red).

A total of 6810 CHD targets were obtained from the GeneCards database. After those with a relevance score >30 had been screened out, a total of 450 targets remained. A total of 162 targets common to DXTMG and CHD were identified using the Venny online tool (Figure 2).

### Determination of targets of DXTMG active components and “TCM-compound-target” network construction

**Figure 3:**
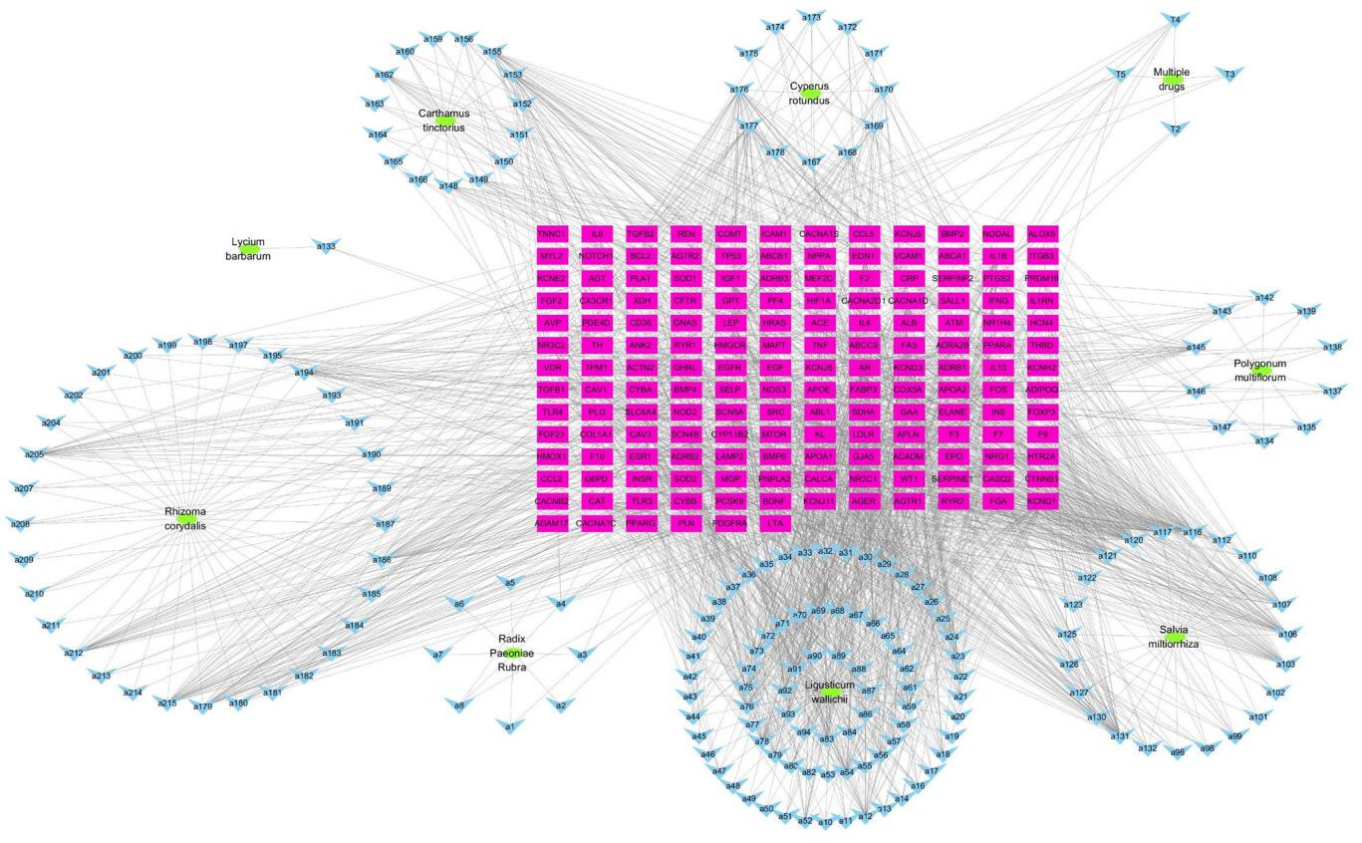
**“TCM-compound-target” network diagram.** (Green ovals represent TCM; blue inverted triangles represent compound; red rectangles represent targets).

The data were visualized by Cytoscape 3.7.2 software. A “TCM-compound-target” network (Figure 3) was constructed, and its nodes were analyzed. The compounds with the top 5 Degrees were Tanshinone IIA, Neotanshinone C, Neocryptotanshinone II, Miltionone I and Patchoulenone (Supplementary Table 2). These compounds are likely to be the major chemical components of DXTMG with efficacy in the treatment of CHD.

### PPI network analysis

**Figure 4:**
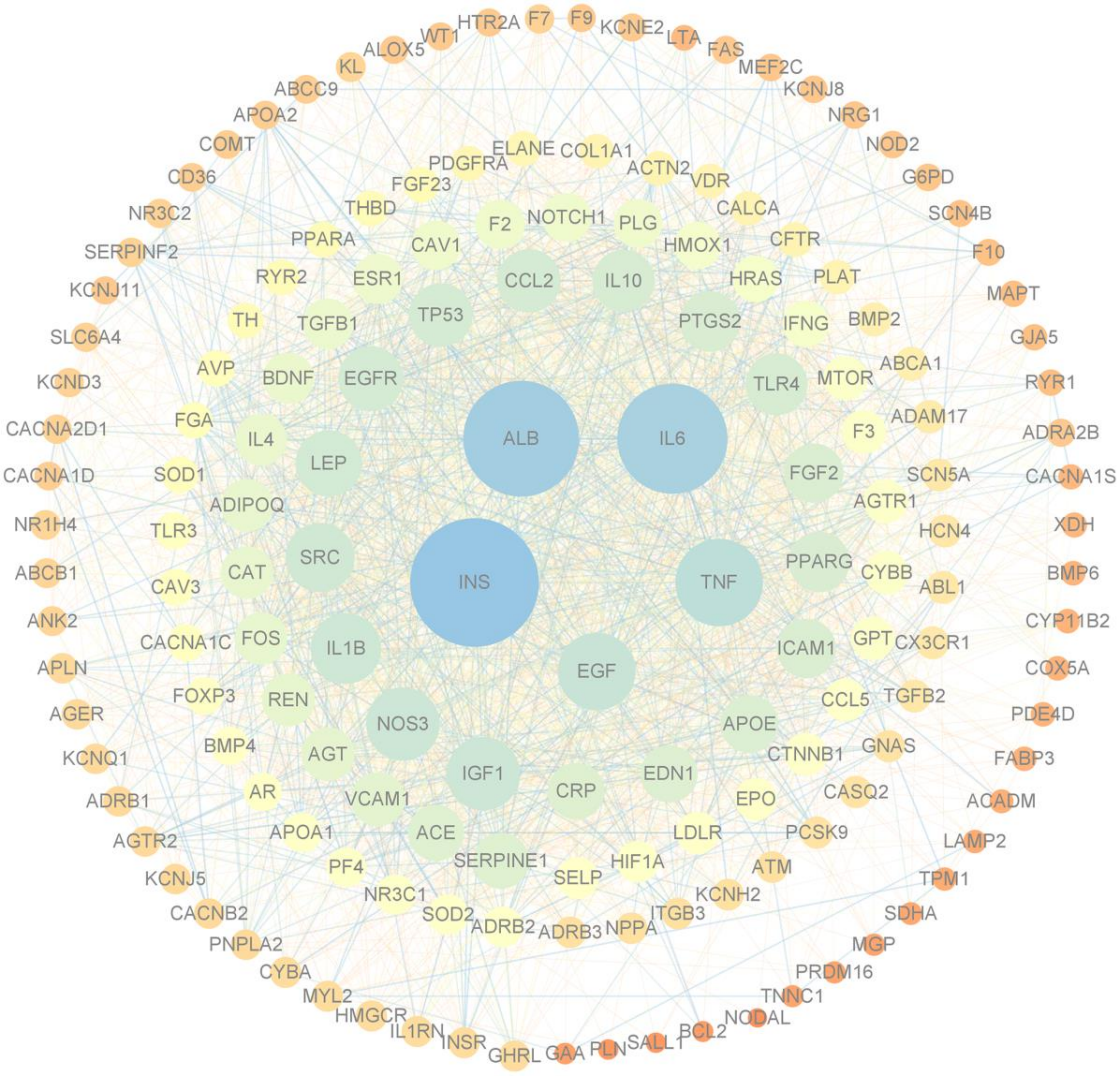
**PPI network of targets of DXTMG and CHD.** (The sizes and colors of the nodes and lines are illustrated from large to small and blue to red in descending order of degree values).

The 162 CHD target proteins of DXTMG were imported into the STRING database and resulting data were imported into Cytoscape 3.7.2 software. A PPI network diagram was constructed (Figure 4). Core targets with the top 4 Degrees were INS, Alb, IL-6 and TNF.

### GO enrichment and KEGG pathway enrichment analysis

**Figure 5:**
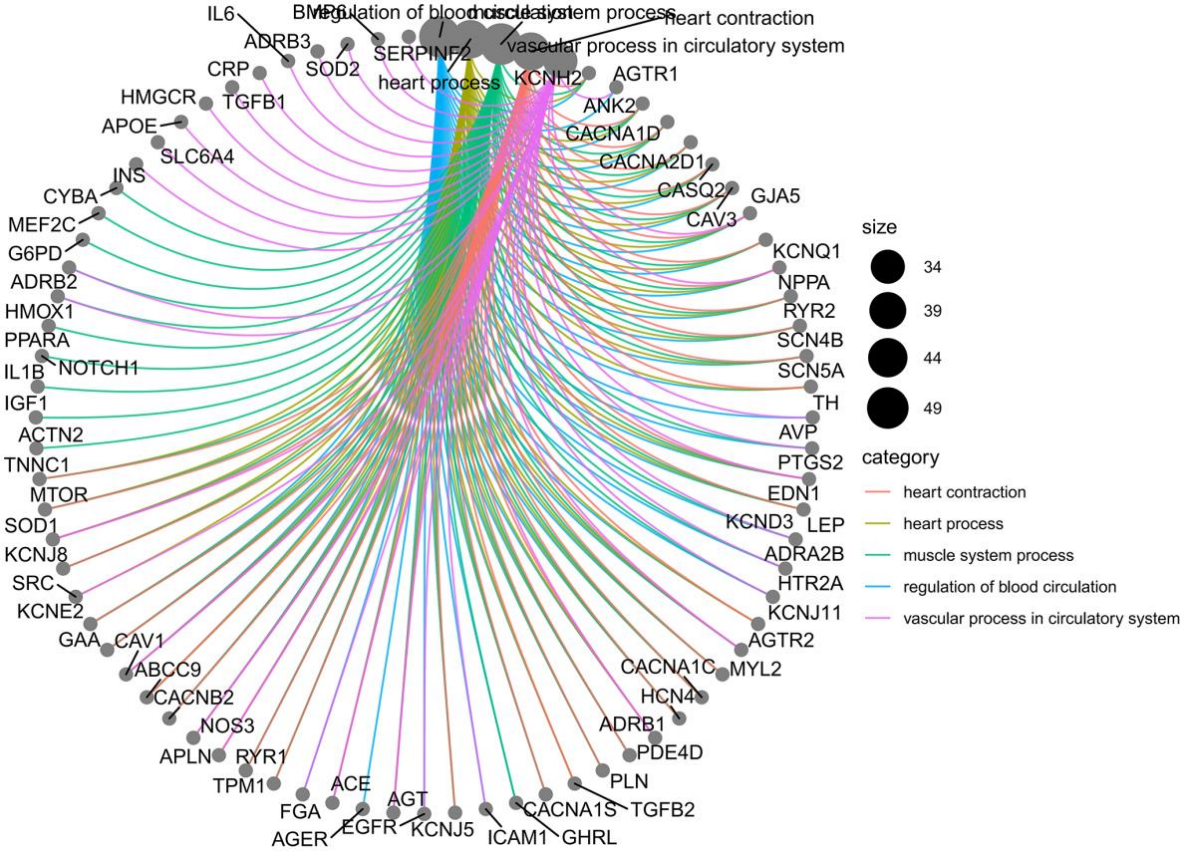
Relationship between BP and targets.

**Figure 6:**
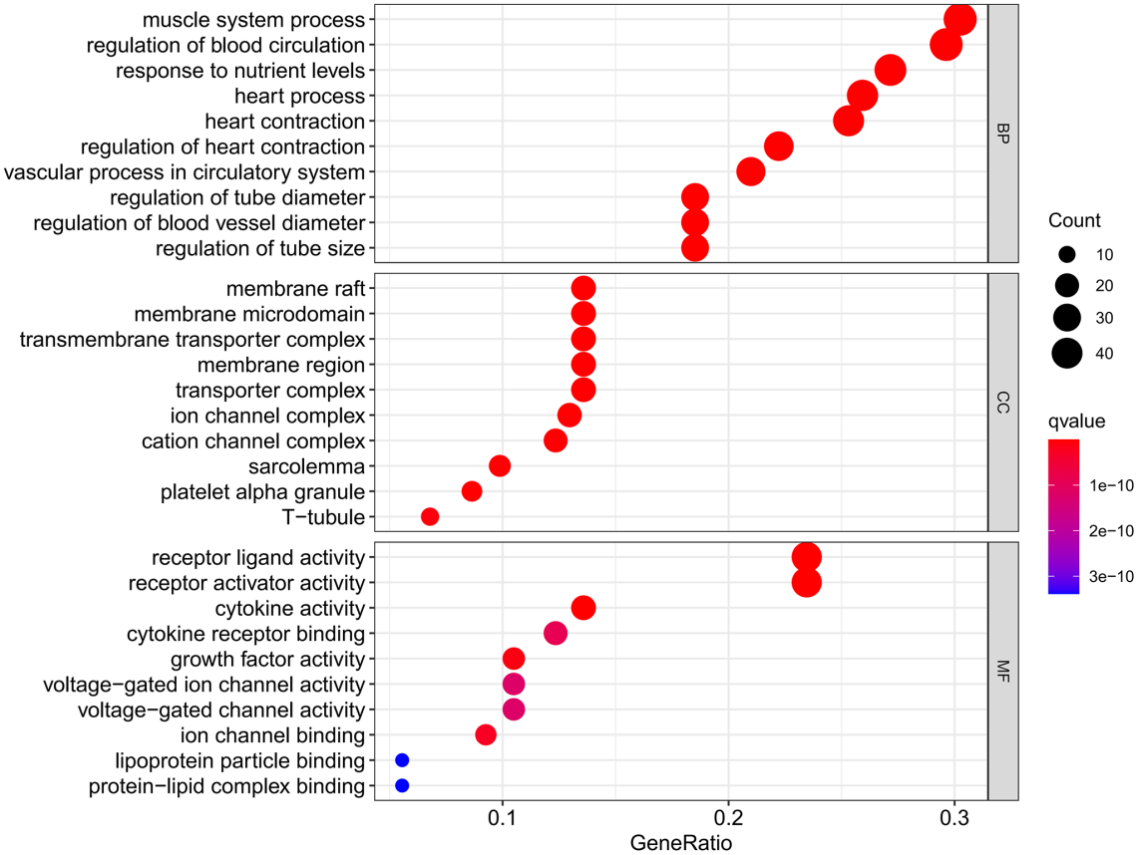
GO analysis results.

The results of GO enrichment analysis showed that there were 3365 GO pathways in total, including 3049 pathways of BP (heart and circulatory system), 109 pathways of CC (cation channels and membrane rafts) and 207 pathways of MF (receptor ligand and activator). The relationships between BP and targets are shown in Figure 5, and the GO analysis results of BP, CC and MF are shown in Figure 6.

**Figure 7:**
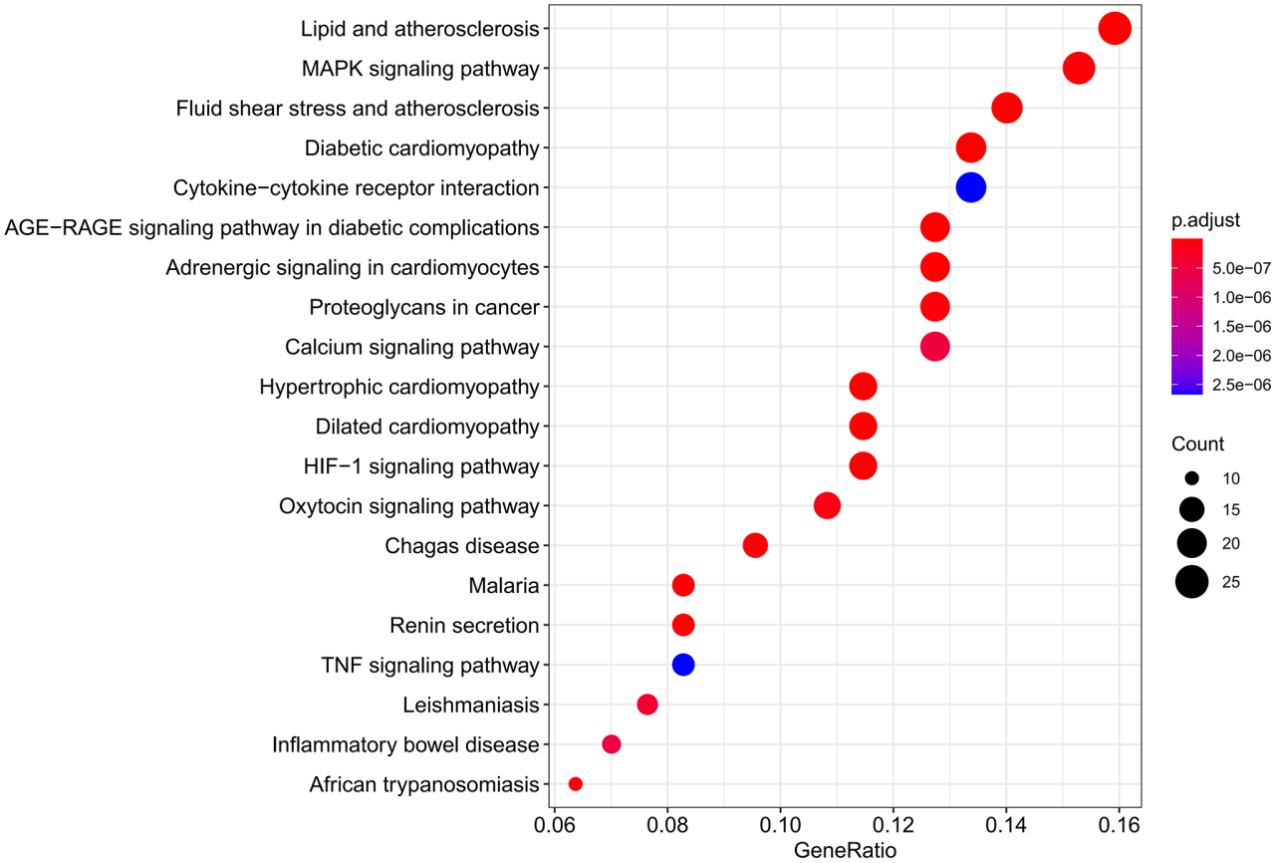
Results of KEGG pathway enrichment analysis.

A total of 137 KEGG pathways were obtained. DXTMG mainly regulated the Lipid and atherosclerosis, MAPK signaling pathway, Fluid shear stress and atherosclerosis, and Diabetic cardiomyopathy. The top 20 KEGG pathways are shown in Figure 7.

### Molecular docking

Molecular docking was conducted for core compounds: Tanshinone IIA, Neotanshinone C, Neocryptotanshinone II, Miltionone I, and Patchoulenone, along with core targets: INS, Alb, IL-6, and TNF. The binding energies were <−7 kcal/mol, indicating a strong binding capacity between the compounds and targets. Among them, the binding strength between TNF-Neocryptotanshinone II was the highest, followed by TNF-Patchoulenone and TNF-Tanshinone IIA. The binding site of TNF-Neocryptotanshinone II is a hydrophobic binding site, with residues forming hydrophobic interactions including Leu481, Phe480, Val482, Phe319, Leu320, Val321, etc. Additionally, the 2D diagram illustrates that the ligand’s hydroxyl group can form a hydrogen bond with Leu320, thereby significantly stabilizing its binding. In contrast, the binding site of TNF-Patchoulenone is hydrophilic, with hydrophilic residues interacting with ligands such as LYS186, LYS191, and hydrophobic residues like TYR254, PHE298. For TNF-Tanshinone IIA, the binding site presents as amphiphilic, with both hydrophilic and hydrophobic characteristics. Hydrophobic residues encompass Ile378, Val377, while hydrophilic residues include Asn368, Glu454, etc.

**Figure 8:**
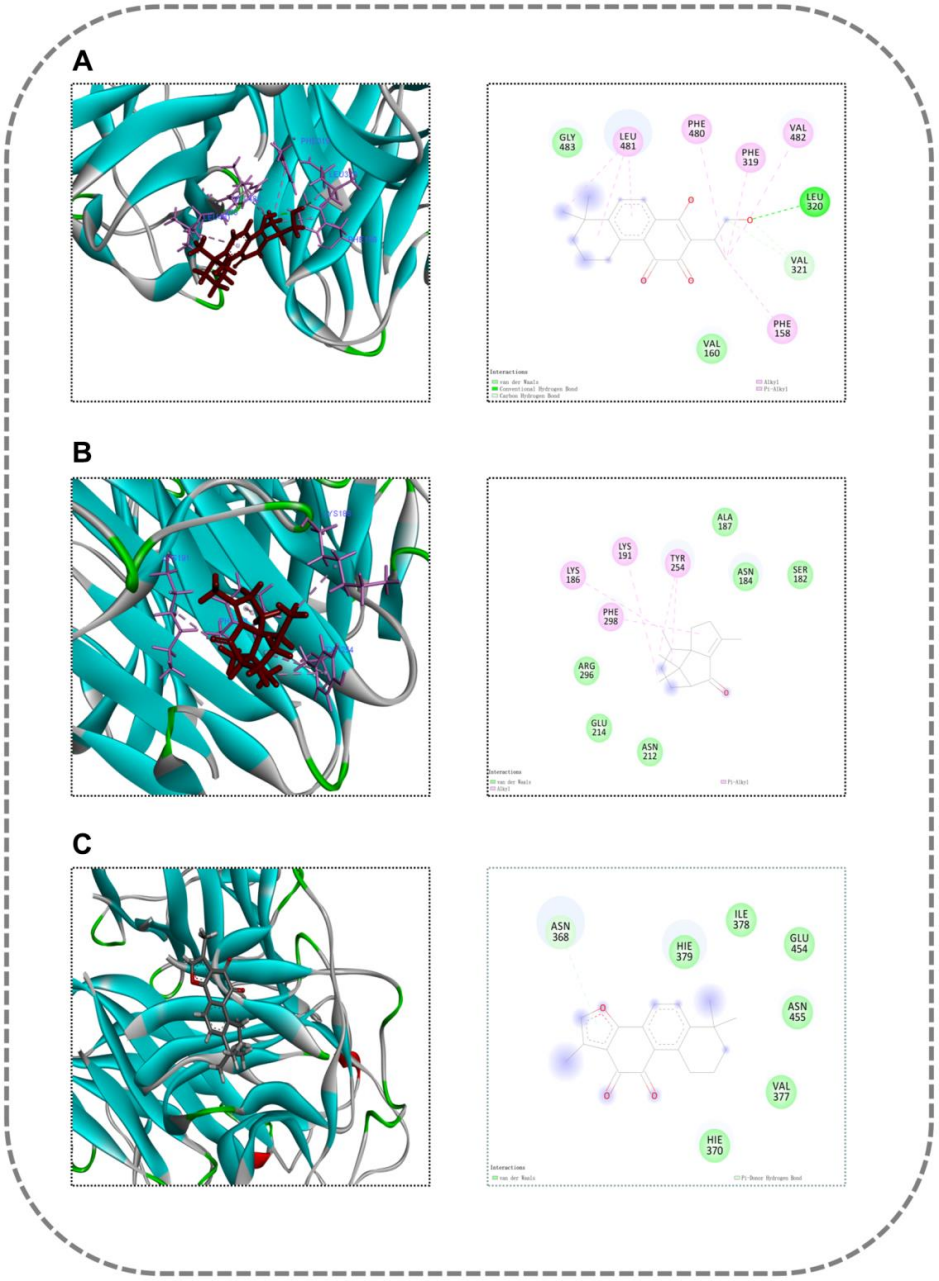
**Molecular docking diagram.** (**A**) TNF-Neocryptotanshinone II; (**B**) TNF-Patchoulenone; (**C**) TNF-Tanshinone IIA.

The molecular docking results of the compounds and target proteins are detailed in Supplementary Table 3, and the molecular docking diagram is depicted in Figure 8.

### Molecular dynamics simulation

Based on binding energy results, molecular dynamics simulation was performed for TNF-Neocryptotanshinone II, TNF-Patchoulenone, and TNF-Tanshinone IIA. The root-mean-square deviation (RMSD), radius of gyration (Rog), root-mean-square fluctuation (RMSF) curve and binding free energy were calculated.

**Figure 9:**
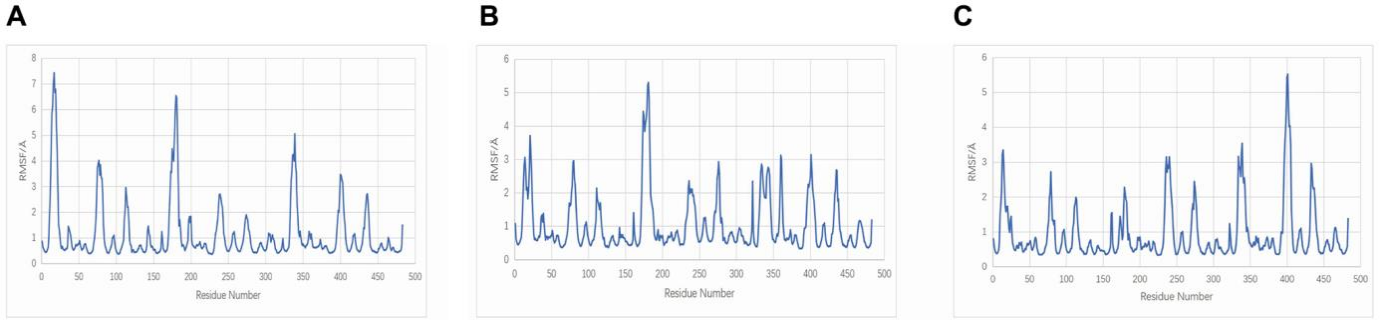
**RMSD plot during molecular dynamics simulations.** (**A**) TNF-Neocryptotanshinone II; (**B**) TNF-Patchoulenone; (**C**) TNF-Tanshinone IIA.

The RMSD curve represented variations in protein conformation. The results show that the RMSD fluctuations are relatively gentle, and the RMSD fluctuations of the TNF-Neocryptotanshinone II protein are relatively gentle, especially after 80 ns, such that the RMSD is stable around 4~4.5 Å. The fluctuations in RMSD of TNF-Patchoulenone and TNF-Tanshinone IIA proteins are relatively gentle, especially after 100 ns, such that the RMSD is stable around 3~3.5 Å. This result shows that the binding of the small molecule to the receptor protein does not lead to a sustained and significant change in its conformation. The RMSD results are shown in Figure 9.

**Figure 10:**
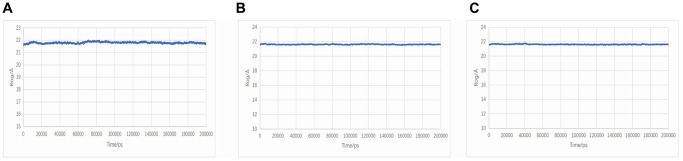
**Rog plot during molecular dynamics simulations.** (**A**) TNF-Neocryptotanshinone II; (**B**) TNF-Patchoulenone; (**C**) TNF-Tanshinone IIA.

The Rog curve displayed the compactness of the overall protein structure. The radius of gyration of TNF-Neocryptotanshinone II and TNF-Patchoulenone slightly decreased, indicating that the binding of small molecules to the receptor may make the receptor protein structure more compact. However, the radius of gyration of TNF-Tanshinone IIA did not change significantly, indicating that the binding of the small molecule to the receptor had no effect on the protein conformation. The ROG results are shown in Figure 10.

**Figure 11:**
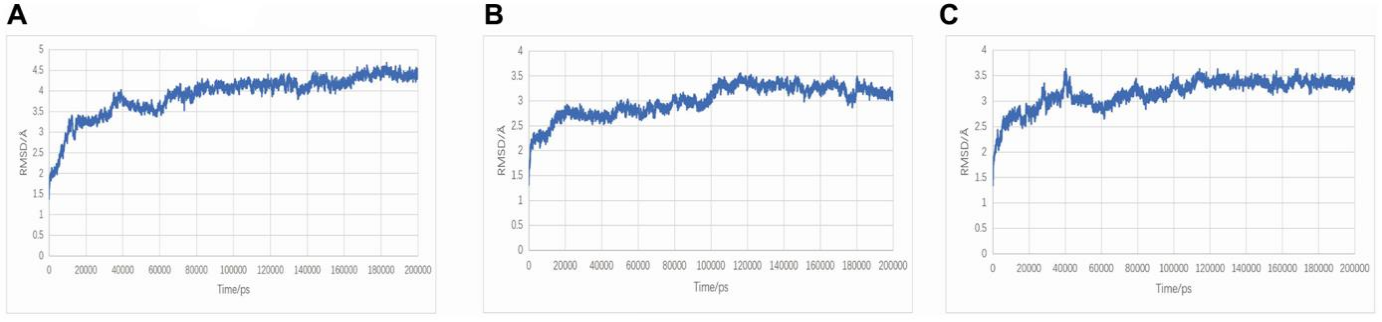
**RMSF plot during molecular dynamics simulations.** (**A**) TNF-Neocryptotanshinone II; (**B**) TNF-Patchoulenone; (**C**) TNF-Tanshinone IIA.

The RMSF curve displays variations in the conformation of amino acid residues. Several smaller regions of the protein core domain of TNF-Neocryptotanshinone II, TNF-Patchoulenone, and TNF-Tanshinone IIA have greater flexibility than other regions, and these regions include 0–25, 70–80, 170–180 and 390–410; most of these regions are loop-based, so they have high flexibility. The RMSF results are shown in Figure 11.

**Figure 12:**
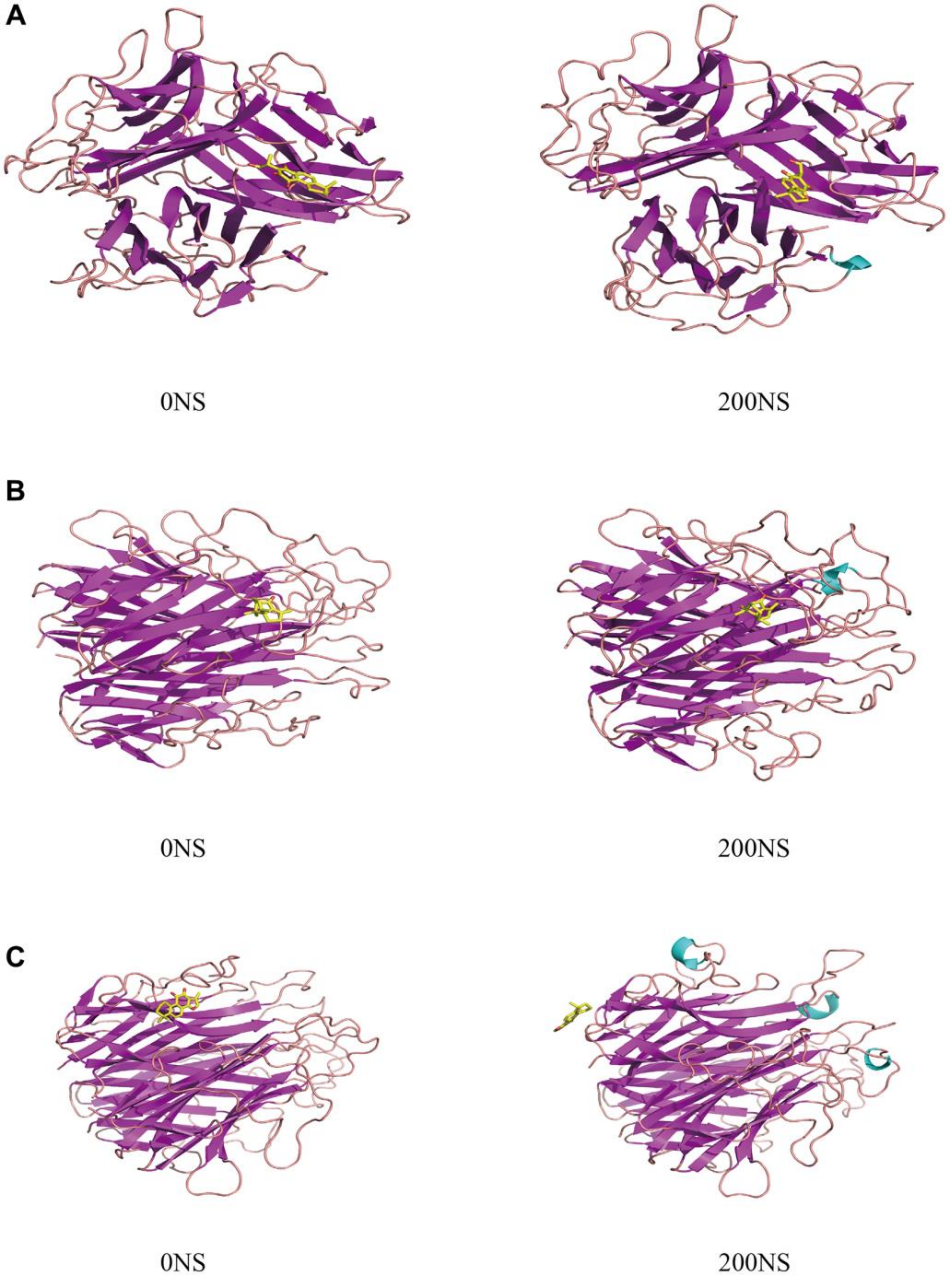
**The protein conformational changes results.** (**A**) TNF-Neocryptotanshinone II; (**B**) TNF-Patchoulenone; (**C**) TNF-Tanshinone IIA.

Protein conformational changes: After a 200-ns molecular dynamics simulation process, TNF and Neocryptotanshinone II form a tight bond. The kinetics did not alter the binding site or the binding stability of the small molecule, demonstrating enhanced binding to the receptor protein. The binding sites of TNF and Patchoulenone exhibited slight alterations, yet the small molecule consistently maintained tight binding to the receptor protein without detachment, indicating improved binding. Conversely, the binding sites of TNF and Tanshinone IIA underwent significant changes, and the small molecules exhibited a tendency to detach, indicating poor binding to the receptor protein, consistent with the binding free energy data. The results of the protein conformational changes are shown in Figure 12.

### HE staining results

**Figure 13:**
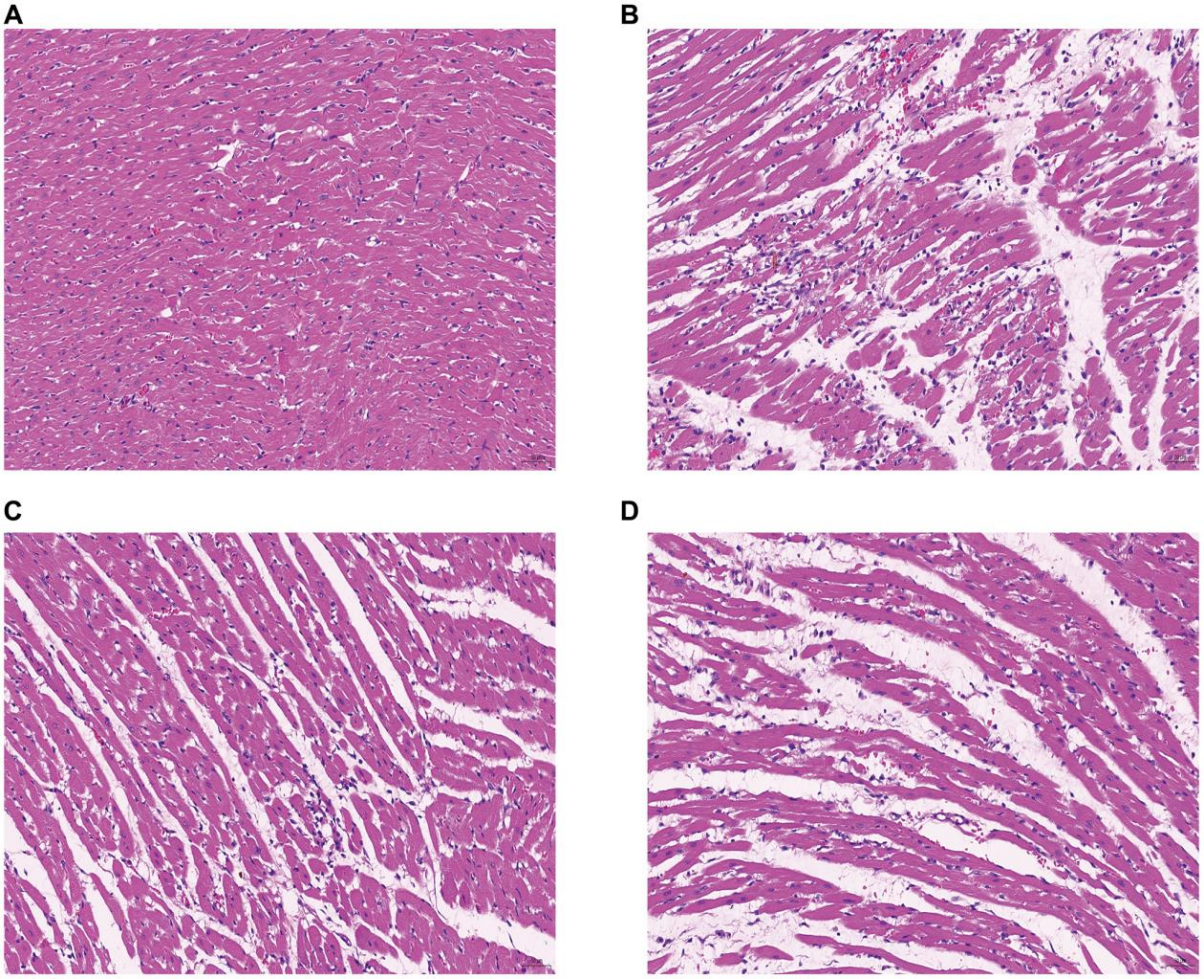
**Morphology of rat cardiomyocytes in each group (HE staining, ×20).** (**A**) Normal group (**B**) Model group (**C**) DXTMG group (**D**) Propranolol group.

As shown in Figure 13, the cardiac tissue of the control group exhibited uniformly colored cardiac myofibers, distinct cell demarcations, clear transverse striations of cardiomyocytes, and well-defined bright and dark areas, with no abnormalities observed in the interstitium. Furthermore, no obvious necrosis or inflammatory cell infiltration was detected. In contrast, the model group displayed multifocal necrosis of cardiomyocytes, cell fragmentation, nucleus fragmentation and lysis, along with increased inflammatory cell infiltration in the interstitium. In the DXTMG group, there was a small amount of inflammatory cell infiltration in the interstitium.

### Effects of DXTMG on serum LDH and cTnI levels in rats with acute myocardial ischemia

**Figure 14:**
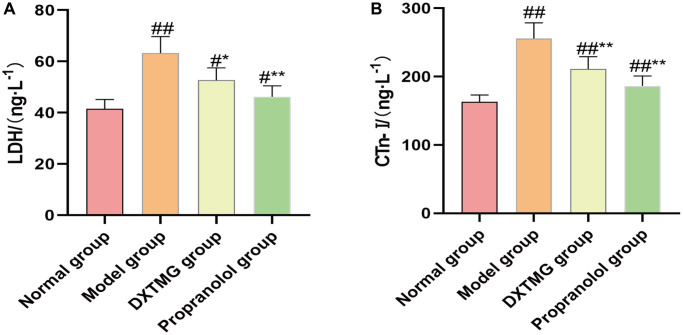
**Plasma LDH and CTn-I levels in the normal group, the model group, the DXTMG group and the propranolol group.** (**A**) lactate dehydrogenase (LDH); (**B**) Cardiac troponin I (CTn-I). Data are presented as the mean ± S.D. (*n* = 8). ^##^*P* < 0.01 vs. Normal group; ^#^*P* < 0.05 vs. Normal group; ^**^P < 0.01 vs. Model group; ^*^P < 0.05 vs. Model group.

As shown in Figure 14, serum LDH and cTnI levels were elevated in the model group compared to the normal group (*P* < 0.01). Conversely, serum LDH and cTnI levels were reduced in the DXTMG group compared to the model group (*P* < 0.05, *P* < 0.01).

### MASSON staining results

**Figure 15:**
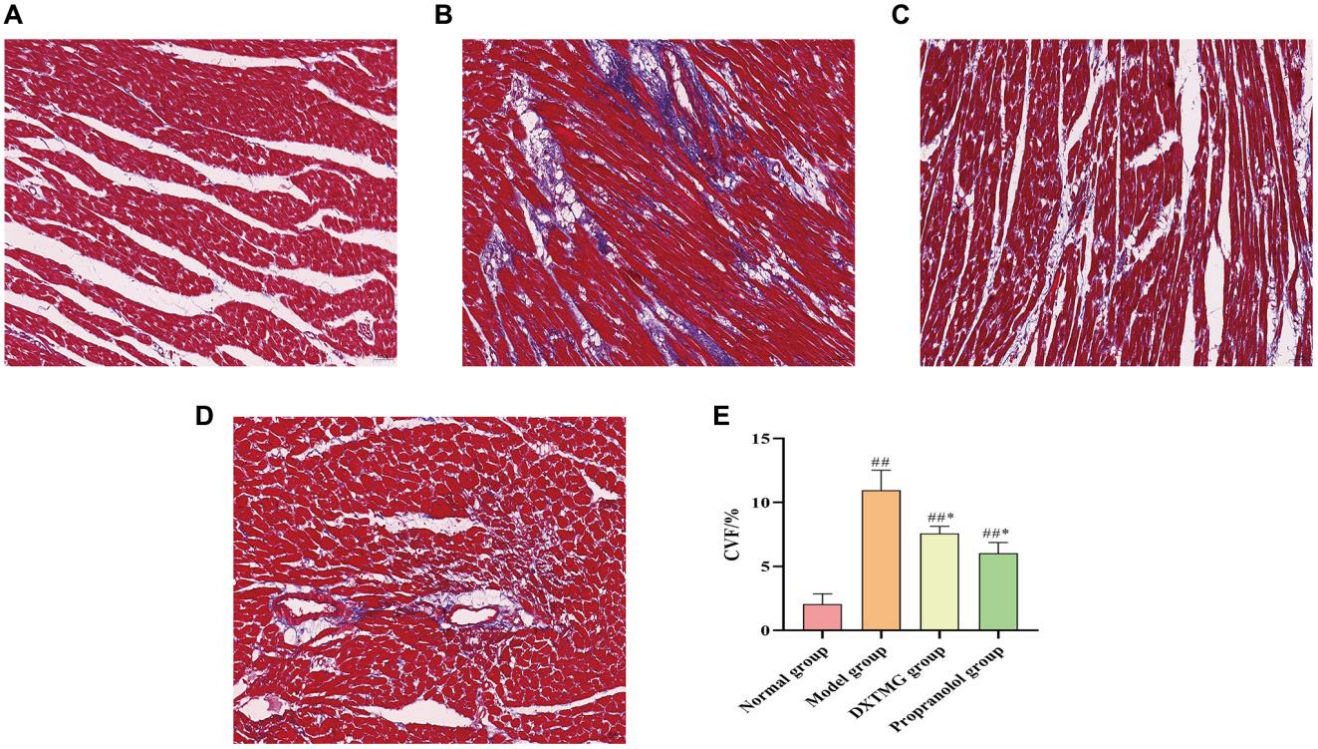
**Morphology of rat cardiomyocytes in each group (Masson staining, ×20).** (**A**) Blank group; (**B**) Model group; (**C**) DXTMG group; (**D**) Propranolol group; (**E**) Comparison of CVF in rats of various groups.

As shown in Figure 15, myocardial fibers in the Normal group were neatly arranged, with clearer transverse lines and no obvious collagen deposition. In contrast, the model group exhibited disordered myofiber arrangement, fibrotic interstitium, and severe collagen deposition. However, in the DXTMG group, the myofiber arrangement was partially disordered, with a small amount of collagen deposition observed. The area of collagen deposition in each group was quantified, revealing a reduction in the DXTMG group compared to the model group (*P* < 0.05).

### Effects of DXTMG on SOD, GSH-Px activity and MDA levels in myocardial tissues of rats with acute myocardial ischemia

**Figure 16:**
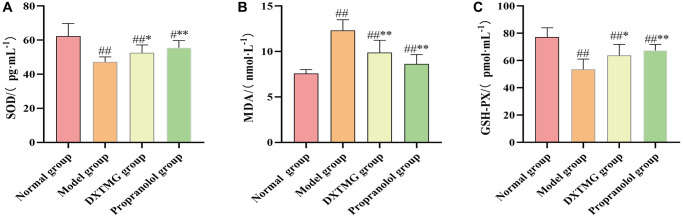
**Myocardium SOD, MDA, and GSH-PX levels in the normal group, model group, DXTMG group, and propranolol group.** (**A**) Rat Superoxide Dismutase (SOD); (**B**) Malondialdehyde (MDA); (**C**) Glutathione Peroxidase (GSH-PX). Data are presented as the mean ± S.D. (*n* = 8). ^##^*P* < 0.01 vs. Normal group; ^**^P < 0.01 vs. Model group; *P < 0.05 vs. Model group.

As shown in Figure 16, compared to the Normal group, myocardial tissue SOD and GSH-Px activities in rats of the Model group were decreased (*P* < 0.01), while MDA levels were increased (*P* < 0.01). In comparison to the Model group rats, myocardial tissue SOD and GSH-Px activities in the DXTMG group were increased (*P* < 0.05), and MDA levels were decreased (*P* < 0.01).

### Immunohistochemistry results

**Figure 17:**
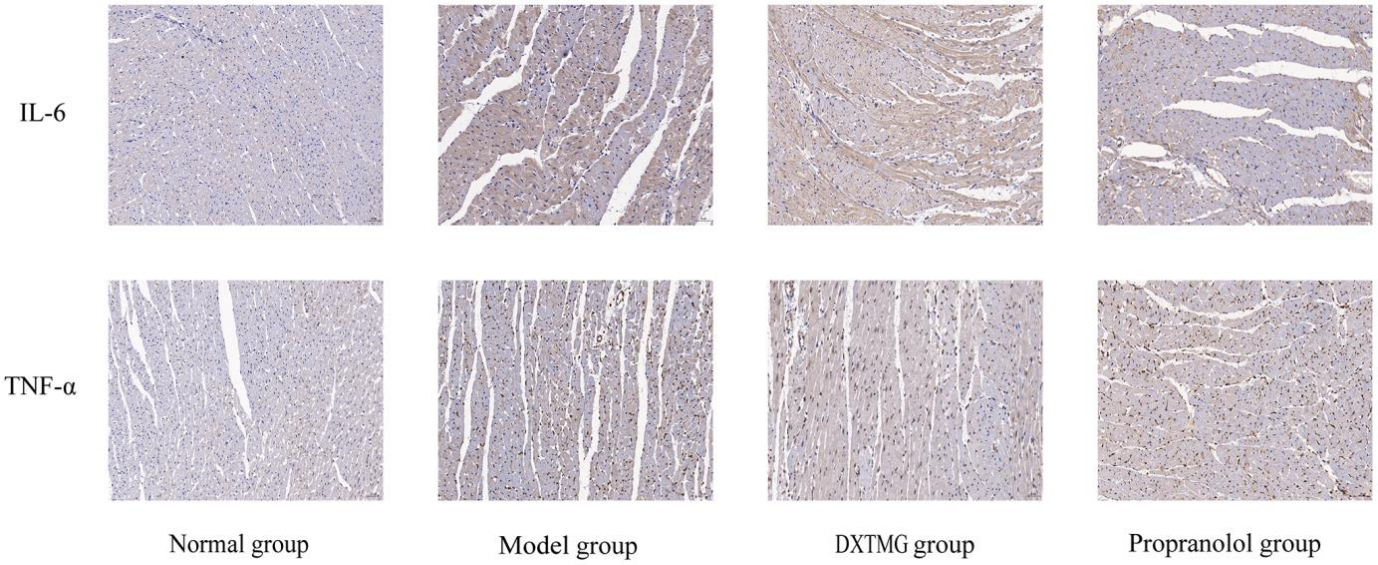
Immunohistochemistry of cellular IL-6 and TNF-α expression in rat cardiomyocytes from 4 groups (×20).

**Figure 18:**
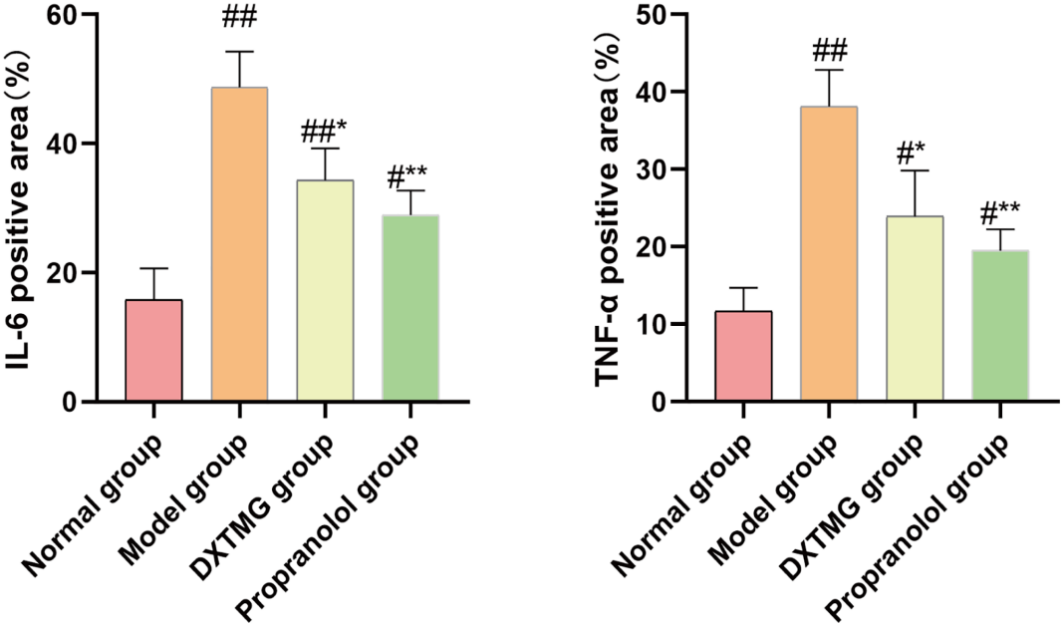
**Comparison of the percentage of positive area of IL-6 and TNF-α in myocardial tissue of rats from 4 groups (** $\bar{x}\pm \text{s}$ **, *n* = 3).**

As shown in Figures 17 and 18, a small number of brownish-yellow positive particles appeared in the cytoplasm of myocardial tissue cells in the Normal group rats. In comparison to the Normal group, the relative expression of IL-6 and TNF-α positive cells in the myocardial tissue of Model group rats increased (*P* < 0.01). Conversely, compared to the Model group, the relative expression of IL-6 and TNF-α positive cells in the myocardial tissue of DXTMG group rats decreased (*P* < 0.05).

## DISCUSSION

The current study presents evidence that Tanshinone IIA, Neotanshinone C, Neocryptotanshinone II, Miltionone I, and Patchoulenone are the major compounds of DXTMG with potential pharmacological activity. Previous work at the cellular level has established that Tanshinone IIA greatly relieves myocardial inflammatory infiltration and oxidative damage caused by myocardial ischemia-reperfusion injury in rats and reduces leakage of lactate dehydrogenase and production of MDA, thereby protecting against myocardial ischemia-reperfusion injury [19]. Tanshinone IIA has an estrogen-like effect resulting in protein kinase B (Akt) activation and suppression of cardiomyocyte apoptosis on activation by insulin-like growth factor II (IGF-II). This suggests a role for Tanshinone IIA as a potential selective estrogen receptor modulator in the prevention of myocardial apoptosis and treatment of cardiovascular diseases [20]. Moreover, Li et al. [21] demonstrated a role for Tanshinone IIA in suppressing mitogen-activated protein kinase (MAPK) and down-regulating c-fos expression, thus reducing intimal hyperplasia and vascular smooth muscle cell proliferation in rats with carotid artery balloon injury. Neocryptotanshinone suppresses lipopolysaccharide-induced inflammatory factors, including TNF-α and IL-6, in rats. Neocryptotanshinone also reduces lipopolysaccharide-induced production of nitric oxide [22]. The pharmacological and computer-based investigations of Maione et al. [23] demonstrated a concentration-dependent activity of Neocryptotanshinone in inhibiting platelet aggregation and antagonizing G protein-coupled P2Y12 platelet ADP receptor.

The PPI analysis in this study identified INS, ALB, IL-6, and TNF as major targets of DXTMG in CHD treatment. Previous studies have established that insulin resistance (IR) serves as a predisposing factor for cardiovascular diseases, including CHD [24]. IR leads to elevated levels of inflammatory cytokines, increased expression of adhesion molecules, imbalanced vascular regulation, and vascular endothelial injury, ultimately culminating in atherosclerosis [25]. INS plays a crucial role in enhancing glucose uptake and utilization, thereby safeguarding the ischemic-hypoxic myocardium. Elevated blood glucose levels pose a risk factor for cardiovascular diseases, and the hypoglycemic effect of INS is pivotal in blood glucose regulation [26]. High levels of INS augment angiotensin II through the renin-angiotensin system and promote the production of reactive oxygen species, thereby facilitating inflammatory and oxidative stress responses [27]. Normal levels of albumin inhibit platelet activation, aggregation, and apoptosis of vascular endothelial cells, thus reducing the risk of CHD [28]. The accumulation of inflammatory cells around damaged vascular tissues may promote plaque formation via the secretion of inflammatory mediators, making inflammatory markers significant risk factors for CHD [29, 30]. Scheller et al. [31] demonstrated a positive correlation between IL-6 levels and the incidence of severe coronary artery lesions. IL-6 promotes macrophage activation and formation of foam cells, stimulates the expression of macrophage low-density lipoprotein receptors, and triggers matrix metalloproteinases to destabilize atheromatous plaques, leading to plaque rupture [32]. Vascular endothelial injury is known to stimulate inflammatory receptors and the release of inflammatory factors, such as TNF-α and IL-6, followed by thrombosis and plaque rupture [33]. The suppression of smooth muscle cell proliferation, which correlates with down-regulated expression of TNF-α and IL-6, may enhance the stability of atherosclerotic plaques [34]. Additionally, IL-6 and TNF-α, as the main pro-inflammatory markers in myocardial injury, induce the recruitment of more neutrophils, monocytes, and lymphocytes from peripheral blood to the injured myocardium, initiating an inflammatory cascade and stimulating a more intense inflammatory response, leading to cardiac hypertrophy, hyperfibrosis, and apoptosis [35].

In this experiment, a rat myocardial ischemia model was established by pretreating with DXTMG via gavage followed by injection of 85 mg·kg^−1^ ISO for two consecutive days starting on the 6th day. The protective effect and mechanism of DXTMG on myocardial ischemia were investigated by comparing the results of HE staining, MASSON staining, immunohistochemistry, and ELISA. Currently, dorsal injection of ISO in rats is one of the commonly used models of myocardial ischemic injury. ISO leads to rapid generation and aggregation of a large number of free radicals through its own oxidation, thus inducing myocardial ischemia and hypoxia. This method can simulate the alterations seen in acute myocardial ischemia [36].

cTnI is a regulatory protein specific to myocardial tissue and plays a pivotal role in myocardial contraction [37]. During myocardial ischemia, increased cell membrane permeability allows cTnI and LDH, among other substances, to enter the bloodstream through the cell membrane. These are sensitive and specific serum markers of myocardial cell injury [38].

The results of this experiment showed that serum LDH and cTnI levels were abnormally elevated in the model group rats, indicating obvious myocardial injury. However, DXTMG significantly reduced the release of LDH and cTnI in cardiomyocytes. Additionally, HE staining of rat myocardium in the model group revealed multifocal necrosis of cardiomyocytes, cell fragmentation, cytoplasmic fragmentation and lysis, and increased inflammatory cell infiltration in the interstitium. MASSON staining showed disorganized arrangement of myofibrils, fibrotic changes in the interstitium, and severe collagen deposition. Immunohistochemistry results demonstrated elevated the percentage of IL-6 and TNF-α positive areas; however, DXTMG reduced the percentage of IL-6 and TNF-α positive areas, and attenuated the release of LDH and cTnI in rat myocytes. DXTMG could reduce the expression of IL-6 and TNF-α in rat myocardium and attenuate the inflammatory reaction and fibrotic changes in myocardial tissue, suggesting that anti-inflammation and anti-myocardial fibrosis may be another important target of DXTMG in attenuating myocardial ischemic injury. SOD reflects the body’s ability to scavenge free radicals, while MDA reflects the degree of cellular damage caused by free radicals [39]. GSH is the main component of the cellular antioxidant defense system, playing a crucial role in maintaining normal cellular metabolism and regulating the body’s immune response [40]. These indicators collectively constitute the body’s defense system against oxidative damage caused by free radicals, which is essential for maintaining oxidative/antioxidant homeostasis in the body. The present experiment demonstrated that DXTMG significantly increased SOD and GSH levels, reduced MDA levels, reestablished the free radical balance, and scavenged oxygen radicals through antioxidant enzymes. This restoration of cardiac function alleviated oxidative damage to cardiomyocytes. In conclusion, the protective effect of DXTMG on isoprenaline-induced acute myocardial ischemia in rats may be related to its scavenging of free radicals, enhancement of antioxidant enzymes, inhibition of inflammatory factor expression, and reduction of inflammatory response.

The current study used KEGG pathway enrichment analysis to demonstrate that fluid shear stress and atherosclerosis, the hypoxia-inducible factor-1 (HIF-1) signaling pathway, adrenergic signaling in cardiomyocytes and the AGE-RAGE signaling pathway in diabetic complications were implicated in the action of DXTMG in CHD treatment. Atherosclerosis is involved in the pathological development of CHD when plaque formation-induced changes in shear stress affect the normal function and phenotype of vascular wall endothelial cells [41]. It has been established that low shear stress up-regulates expression of intercellular adhesion molecule-1 on the endothelial cell surface, promoting activation of endothelial cells [42]. A further consequence is phosphorylation of platelet endothelial cell adhesion molecule-1, activating the MAPK pathway and worsening inflammation to promote CHD. HIF-1, a hypoxia-induced DNA-binding protein, induces increased expression of HIF-1α during myocardial ischemia and hypoxia, thereby protecting the myocardium [43]. Moreover, HIF-1 induces endothelial cell dysfunction, proliferation, angiogenesis and inflammation, thus playing a role in the pathogenesis of atherosclerosis via various pathways [44]. Via its action on the α1 receptor in vascular smooth muscle, epinephrine promotes vasoconstriction and increases aortic diastolic pressure, thereby raising coronary perfusion pressure [45]. Adrenergic signaling in cardiomyocytes enhances myocardial contractility, resulting in vascular dilation of both heart and liver and vascular contraction of both skin and mucosa, thereby exerting a therapeutic effect on CHD. Activated AGE/RAGE signaling, which enhances inflammation, oxidative stress and vascular smooth muscle cell apoptosis, thus promoting the development of atherosclerosis, plays a significant role in CHD through the above mechanisms [46].

We present results of molecular docking experiments, which revealed stable binding between components of DXTMG and receptor proteins with the binding energy of the TNF-Neocryptotanshinone II interaction being the highest, followed by TNF-Patchoulenone and TNF-Tanshinone IIA. Molecular dynamics simulations showed that the binding of TNF-Neocryptotanshinone II, TNF-Patchoulenone and TNF-Tanshinone IIA was relatively stable.

## CONCLUSION

The current study constructed a “TCM-compound-target” network and PPI network to investigate the role of DXTMG in the treatment of CHD using the approach of network pharmacology. Findings were validated by molecular docking and molecular dynamics simulation. Our findings reveal DXTMG can influence oxidative stress, inflammation response and cardiomyocytes regulation, thereby reducing the occurrence and development of CHD, and exposing avenues for further investigation through animal and clinical experiments.

## Supplementary Materials

Supplementary Tables
