# Supplementary material for: Molecular mechanism of Danxiong Tongmai Granules in treatment of coronary heart disease
Source: Aging (Albany NY). 2024 May 21;16(10):8843–65. doi: 10.18632/aging.205845 (PMC11164497; doi:10.18632/aging.205845)
Supplement: Supplementary Tables [file aging-16-205845-s001.pdf]

SUPPLEMENTARY TABLES

Supplementary Table 1. Active ingredients of DXTMG.

Drug	Active ingredients	No.	Drug	Active ingredients	No.
Radix Paeoniae Rubra	(-)-Catechin	a1	Salvia miltiorrhiza	Isotanshinone II	a111
Radix Paeoniae Rubra	(+)-Catechin	a2	Salvia miltiorrhiza	Isotenulin	a112
Radix Paeoniae Rubra	Albiflorin	a3	Salvia miltiorrhiza	Lithospermate B	a113
Radix Paeoniae Rubra	Beta-Sitosterol	a4	Salvia miltiorrhiza	Lithospermic Acid	a114
Radix Paeoniae Rubra	Catechin	a5	Salvia miltiorrhiza	Magnesium Lithospermate B	a115
Radix Paeoniae Rubra	Epigallocatechin	a6	Salvia miltiorrhiza	Miltionone I	a116
Radix Paeoniae Rubra	Gallocatechin	a7	Salvia miltiorrhiza	Miltirone	a117
Radix Paeoniae Rubra	Lactiflorin	a8	Salvia miltiorrhiza	Monomethyl Lithospermate	a118
Radix Paeoniae Rubra	Paeonol	a9	Salvia miltiorrhiza	Neocryptotanshinone	a119
Ligusticum wallichii	(Z,Z')-Diligustilide	a10	Salvia miltiorrhiza	Neocryptotanshinone II	a120
Ligusticum wallichii	ta-Hydroxybutyryl)-10-Deacetylbac	a11	Salvia miltiorrhiza	Neotanshinone C	a121
Ligusticum wallichii	13-Methyl Pentadecanoic Acid	a12	Salvia miltiorrhiza	Rosmarinine	a122
Ligusticum wallichii	1-Methoxy-2-Methylanthraquinone	a13	Salvia miltiorrhiza	Salonitenolide	a123
Ligusticum wallichii	1-Phenyl-1-Pentanone	a14	Salvia miltiorrhiza	Salvianolicacid B	a124
Ligusticum wallichii	-2,6-Dimethyl-8-(3-Methyl-2-Buten	a15	Salvia miltiorrhiza	Salvinone	a125
Ligusticum wallichii	24-Ethylidene Lophenol	a16	Salvia miltiorrhiza	Salviol	a126
Ligusticum wallichii	3(S)-3-Butyl-4,5-Dihydrophthalide	a17	Salvia miltiorrhiza	Samaderin A	a127
Ligusticum wallichii	3-Carene	a18	Salvia miltiorrhiza	Tanshinone II A	a128
Ligusticum wallichii	droxy-4,5,6,7-Tetrahydro-6,7-Dihy	a19	Salvia miltiorrhiza	Tanshinone II B	a129
Ligusticum wallichii	5,6-Dihydroergosterol	a20	Salvia miltiorrhiza	Tanshinone VI	a130
Ligusticum wallichii	Alpha-Humulene	a21	Salvia miltiorrhiza	Tanshiquinone B	a131
Ligusticum wallichii	Alpha-Phellandrene	a22	Salvia miltiorrhiza	Ursolicacid	a132
Ligusticum wallichii	Alpha-Pinene	a23	lycium barbarum	Betaine	a133
Ligusticum wallichii	Alpha-Terpinene	a24	Polygonum multiflorum	Chrysarobin	a134
Ligusticum wallichii	Alpha-Terpineol	a25	Polygonum multiflorum	Chrysazin	a135
Ligusticum wallichii	Alpha-Terpinolene	a26	Polygonum multiflorum	Citreorosein	a136
Ligusticum wallichii	Beta-Elemene	a27	Polygonum multiflorum	Emodin	a137
Ligusticum wallichii	Beta-Eudesmol	a28	Polygonum multiflorum	Emodin Anthrone	a138
Ligusticum wallichii	Beta-Pinene	a29	Polygonum multiflorum	Fetidine	a139
Ligusticum wallichii	Borneol	a30	Polygonum multiflorum	N-Trans-Feruloyltyramine	a140
Ligusticum wallichii	Butylidenecyclohexane	a31	Polygonum multiflorum	Physcion	a141
Ligusticum wallichii	Caffeic Acid Dimethyl Ether	a32	Polygonum multiflorum	Polygalacic Acid	a142
Ligusticum wallichii	Camphene	a33	Polygonum multiflorum	Polygodial	a143
Ligusticum wallichii	Camphor	a34	Polygonum multiflorum	Questin	a144
Ligusticum wallichii	Choline	a35	Polygonum multiflorum	Resveratrol	a145
Ligusticum wallichii	Chrysanthemaxanthin	a36	Polygonum multiflorum	Rhein	a146
Ligusticum wallichii	chuanxiongzine	a37	Polygonum multiflorum	Tetrahydroxytaxadiene	a147
Ligusticum wallichii	Cibarian	a38	Carthamus tinctorius	1-Heptadecene	a148
Ligusticum wallichii	Citronellol	a39	Carthamus tinctorius	1-Hexadecene	a149
Ligusticum wallichii	Citronellyl Acetate	a40	Carthamus tinctorius	2-Hexanol	a150
Ligusticum wallichii	Cnidilide	a41	Carthamus tinctorius	3-Hexanol	a151
Ligusticum wallichii	Cnidium Lactone	a42	Carthamus tinctorius	Alpha-Onocerin	a152
Ligusticum wallichii	Cocaine	a43	Carthamus tinctorius	Arachidic Acid	a153
Ligusticum wallichii	Dibutyl Phthalate	a44	Carthamus tinctorius	Carthamidin	a154
Ligusticum wallichii	Enanthaldehyde	a45	Carthamus tinctorius	Carvacrol	a155
Ligusticum wallichii	Ethyl Hexadecanoate	a46	Carthamus tinctorius	Clerosterol	a156
Ligusticum wallichii	Ethyl Linoleate	a47	Carthamus tinctorius	Dehydroshikimic Acid	a157
Ligusticum wallichii	Ethyl Palmitate	a48	Carthamus tinctorius	Kaempferol	a158
Ligusticum wallichii	Ethylisoheptadecanoate	a49	Carthamus tinctorius	Lauric Aldehyde	a159
Ligusticum wallichii	Ethylisooctadecanoate	a50	Carthamus tinctorius	Linoleyl Acetate	a160

Ligusticum wallichii	Fenchone	a51
Ligusticum wallichii	Gamma-Terpinene	a52
Ligusticum wallichii	Heptadecanol	a53
Ligusticum wallichii	Hexadecanoic Acid	a54
Ligusticum wallichii	Hexadecanol	a55
Ligusticum wallichii	Humulene	a56
Ligusticum wallichii	Limonene	a57
Ligusticum wallichii	Linalool	a58
Ligusticum wallichii	Linoleic Acid	a59
Ligusticum wallichii	L-Valine-L-Valine Anhydride	a60
Ligusticum wallichii	Menthyl Acetate	a61
Ligusticum wallichii	Methyl Eugenol	a62
Ligusticum wallichii	Methyl Hexadecanoate	a63
Ligusticum wallichii	Methyl Linoleate	a64
Ligusticum wallichii	Methyl Palmitate	a65
Ligusticum wallichii	Methyl Pentadecanoate	a66
Ligusticum wallichii	Methyl Phenyl Carbinol	a67
Ligusticum wallichii	Methyl Phenylacetate	a68
Ligusticum wallichii	Myrcene	a69
Ligusticum wallichii	Naphthalene	a70
Ligusticum wallichii	N-Butyl-2-Ethylbutylphthalate	a71
Ligusticum wallichii	Neocnidilide	a72
Ligusticum wallichii	O-Cresol	a73
Ligusticum wallichii	Octanol	a74
Ligusticum wallichii	O-Ethylphenol	a75
Ligusticum wallichii	Oleic Acid	a76
Ligusticum wallichii	P-Cymene	a77
Ligusticum wallichii	Pentadecanoic Acid	a78
Ligusticum wallichii	Pentadecanol	a79
Ligusticum wallichii	Pentylbenzene	a80
Ligusticum wallichii	Perlolyrine	a81
Ligusticum wallichii	Retinol	a82
Ligusticum wallichii	Sabinene	a83
Ligusticum wallichii	Senkyunolide B	a84
Ligusticum wallichii	Senkyunolide G	a85
Ligusticum wallichii	Senkyunolide K	a86
Ligusticum wallichii	Spathulenol	a87
Ligusticum wallichii	Terpinen-4-Ol	a88
Ligusticum wallichii	Tetradecane	a89
Ligusticum wallichii	Tetramethylpyrazine	a90
Ligusticum wallichii	Thymol	a91
Ligusticum wallichii	Trans-Beta-Farnesene	a92
Ligusticum wallichii	Trans-Caryophyllene	a93
Ligusticum wallichii	Vallesiachotamine	a94
Ligusticum wallichii	Vitamin B1	a95
Salvia miltiorrhiza	1-Hydroxytaxinine A	a96
Salvia miltiorrhiza	6-Hydroxymethyllumazin	a97
Salvia miltiorrhiza	Cryptoxanthin	a98
Salvia miltiorrhiza	danshensu	a99
Salvia miltiorrhiza	danshenxinkun A	a100
Salvia miltiorrhiza	Daphneolone	a101
Salvia miltiorrhiza	Dauricine	a102
Salvia miltiorrhiza	Dehydromiltirone	a103
Salvia miltiorrhiza	Dehydrotremetone	a104
Salvia miltiorrhiza	Dihydroisotanshinone I	a105
Salvia miltiorrhiza	Salvia miltiorrhiza	a106
Carthamus tinctorius	Neocarthamin	a161
Carthamus tinctorius	Neocembrene	a162
Carthamus tinctorius	Quercetin	a163
Carthamus tinctorius	Safranal	a164
Carthamus tinctorius	Sagittariol	a165
Carthamus tinctorius	Stearin	a166
Cyperus rotundus	Alpha-Cyperone	a167
Cyperus rotundus	Beta-Selinene	a168
Cyperus rotundus	Copadiene	a169
Cyperus rotundus	Cyperene	a170
Cyperus rotundus	Cyperol	a171
Cyperus rotundus	Cyperolone	a172
Cyperus rotundus	Isocyperol	a173
Cyperus rotundus	Isokobusone	a174
Cyperus rotundus	Kobusone	a175
Cyperus rotundus	Patchoulenone	a176
Cyperus rotundus	Rotundone	a177
Cyperus rotundus	Sugetriol	a178
Rhizoma corydalis	(+)-Corydaline	a179
Rhizoma corydalis	Allocryptopine	a180
Rhizoma corydalis	Canadine	a181
Rhizoma corydalis	Canaline	a182
Rhizoma corydalis	Columbamine	a183
Rhizoma corydalis	Corchoroside A	a184
Rhizoma corydalis	Corycavine	a185
Rhizoma corydalis	Corydaline	a186
Rhizoma corydalis	Corydalmine	a187
Rhizoma corydalis	Corydamine	a188
Rhizoma corydalis	Corydine	a189
Rhizoma corydalis	Corytuberine	a190
Rhizoma corydalis	Dehydrocorydaline	a191
Rhizoma corydalis	Dehydrocorydalmine	a192
Rhizoma corydalis	Dehydrocostus Lactone	a193
Rhizoma corydalis	Glaucine	a194
Rhizoma corydalis	Isocorydine	a195
Rhizoma corydalis	Isocorynoline	a196
Rhizoma corydalis	Isocorypalmine	a197
Rhizoma corydalis	Isocrotonylpterosin B	a198
Rhizoma corydalis	Methyl Leptol B	a199
Rhizoma corydalis	Norisocorydine	a200
Rhizoma corydalis	Norjuzunal	a201
Rhizoma corydalis	Palmatine	a202
Rhizoma corydalis	Palmidin A	a203
Rhizoma corydalis	Protopine	a204
Rhizoma corydalis	Protoporphyrin	a205
Rhizoma corydalis	Reticulin	a206
Rhizoma corydalis	Reticuline	a207
Rhizoma corydalis	Scoulerine	a208
Rhizoma corydalis	Stylopine	a209
Rhizoma corydalis	Stypandrol	a210
Rhizoma corydalis	Tetrahydrocorysamine	a211
Rhizoma corydalis	Tetrahydropalmatine	a212
Rhizoma corydalis	Thaliporphine	a213
Rhizoma corydalis	Yuanhunine	a214
Rhizoma corydalis	Yuehchukene	a215
Multiple drugs	20-Hexadecanoylingenol	T1

Salvia miltiorrhiza	Salvia miltiorrhiza	a107	Multiple drugs	Caffeicacid	T2
Salvia miltiorrhiza	Salvia miltiorrhiza	a108	Multiple drugs	Caffeicacid	T3
Salvia miltiorrhiza	Isocryptotanshinone	a109	Multiple drugs	Gamma-Sitosterol	T4
Salvia miltiorrhiza	Isocucurbitacin D	a110	Multiple drugs	Gamma-Sitosterol	T5

Supplementary Table 2. Core compounds.

TCM	Compounds	Betweenness centrality	Degree
Salvia miltiorrhiza	Tanshinone IIA	0.043	48
Salvia miltiorrhiza	Neotanshinone C	0.043	48
Salvia miltiorrhiza	Neocryptotanshinone II	0.043	48
Salvia miltiorrhiza	Miltionone I	0.043	48
Cyperus rotundus	Patchoulenone	0.0672	41

Supplementary Table 3. Molecular docking results of compounds and target proteins.

Compounds	Binding energies (kcal/mol)			
	ALB	IL6	INS	TNF
Miltionone I	−9.25 ± 0.29	−8.41 ± 0.02	−8.41 ± 0.02	−11.68 ± 1.28
Neocryptotanshinone II	−10.43 ± 0.56	−10.50 ± 0.20	−10.43 ± 0.15	−14.09 ± 0.13
Neotanshinone C	−10.19 ± 0.86	−8.69 ± 0.08	−8.93 ± 0.41	−13.05 ± 0.32
Patchoulenone	−9.98 ± 0.16	−9.61 ± 0.03	−9.60 ± 0.10	−13.24 ± 0.38
Tanshinone IIA	−8.73 ± 0.20	−8.80 ± 0.01	−8.80 ± 0.02	−13.14 ± 0.06
